# Malignant Pustule

**Published:** 1854-10

**Authors:** E. D. Ayres

**Affiliations:** East Creek, N. Y.


*Malignant Pustule.* By E. D. Ayres, M. D., East Creek, N. Y.—The description of "*A peculiar form of Malignant Inflammation of the Lips and Face, resembling Malignant Pustule,*" by Prof. Parker, in the May number of your Journal, attracted my attention, from the close analogy borne by some of the cases therein described, to an affection which I have several times encountered within the last year. As the description given by Prof. Parker is so applicable to the disease as I have met it, the difference in the treatment, and the fatality of the disease, are the only objects of this communication.

The *first case* that I saw, occurred in the practice of a neighboring physician, and from its malignant type, alarmed his friends, and counsel was desired. I was sent for, and arrived in the evening of the 10th of September last. I found the patient, Mr. B——n, 31 years of age, a farmer of temperate habits and good constitution, with the inferior lip greatly swollen, everted, and of a livid hue, the lividity extending to the mouth and fauces, and the tumefaction to the upper lip, and downwards to the pomum Adami. The tongue was moist; there was considerable dyspnœa, and difficulty of deglutition. The pulse was rapid, but small and fluttering; and the patient had been greatly alarmed, but on the subsidence of the pain had become composed. The attending physician was not present, but from the urgency of the case, I was induced to lay freely open the tumefied lip, and ordered yeast poultice to be applied. I followed this immediately with hot brandy toddy, which was directed to be continued with a powder of two grains of quinine, and four grains of carb. ammonia, given once every two hours, and calomel, two grains, every four hours. The affection had made its appearance in the form of a pustule, some three days previously, and, as in Prof. Parker's first case, was situated just below the vermillion border. It was attended with a burning, smarting sensation, which, as the livid, tumefied areola made its appearance, changed to a steady, intense pain. It was now a hard tumor, without pain, and almost insensible to the touch, resembling at once, erysipelas and carbuncle, and differing essentially from both. The treatment as above directed, was continued thirty-six hours, with decided benefit, the patient evidently gaining strength. There was now added a few grains of the pulv. Doveri to the calomel, as somewhat severe catharsis had supervened.

This treatment was continued twelve hours longer, when slight ptyalism being apparent, it was suspended, as also the brandy. Beef tea, nourishing broths, the quinine and ammonia, were administered for twenty-four hours longer, when the patient was considered beyond danger. He rapidly recovered on nourishing diet.

The *second case*, Mr. C——r, by occupation a carpenter, 35 years of age, temperate, had previously enjoyed good health, was attacked with symptoms similar to the first case. The dyspnœa and dysphagia were not so prominent, and the pustule had made its appearance below the angle of the mouth, on the right side. The tumefaction confined itself, principally, to that side of the face and throat. The period of incubation was longer, the pustule having made its appearance some five days before the occurrence of alarming symptoms. The pulse was 64 and small, and the tendency to death by asthenia, marked. This case was treated like the first, but rather more vigorously; he took three grains of quinine and six of carb. ammonia, every two hours, for the first twenty-four hours. The alarming symptoms subsided in 72 hours, and he resumed his labor in eight days from the time of the first attack of distressing symptoms, and has since remained in good health.

I have seen, during the past winter and spring, three other cases, exactly similar to the above, and have heard of several deaths occurring in the practice of neighboring physicians, from a disease bearing the same description, and doubtless the same disease. But in no case can I learn that death followed the early and vigorous application of the tonic, stimulant, and it may be antiseptic plan.—*New York Journal of Medicine.*

